# Transient right bundle branch block (RBBB) and S1Q3T3 in a transit passenger due to acute pulmonary embolism- a case report

**DOI:** 10.1093/omcr/omae165

**Published:** 2025-01-18

**Authors:** Haris Iftikhar, Doaa Sabir, Mavia Najam, Ahmed Noor, Shahzad Anjum

**Affiliations:** Emergency Medicine, Hamad General Hospital, Al Rayyan Road, P.O. Box 3050, Doha, Qatar; Emergency Medicine, Hamad General Hospital, Al Rayyan Road, P.O. Box 3050, Doha, Qatar; Department of Medical Education, Hamad Medical Corporation, Ahmed Bin Ali St, Doha, Qatar; Emergency Medicine, Hamad General Hospital, Al Rayyan Road, P.O. Box 3050, Doha, Qatar; Emergency Medicine, Hamad General Hospital, Al Rayyan Road, P.O. Box 3050, Doha, Qatar

**Keywords:** embolism, pulmonary, RBBB, transit passenger, head injury, syncope

## Abstract

Intermittent or transient right bundle branch block (RBBB) can occur in various clinical situations but is rarely described in acute pulmonary embolism. We present a unique case involving a 57-year-old male who experienced a syncopal episode during transit. He displayed signs of a transient right bundle branch block (RBBB) and S1Q3T3 on the initial EMS ECG, which reverted to normal ECG later. This case highlights the significance of recognizing transient RBBB and S1Q3T3 patterns as potential markers of acute PE. Anticoagulation therapy is the mainstay of PE treatment, but other modalities like thrombolysis and surgical embolectomy can be used in selected cases.

## Introduction

Acute pulmonary embolism (PE) is a potentially life-threatening condition resulting from the obstruction of pulmonary arteries by emboli, usually originating from deep venous thrombosis. Risk factors for developing PE include major surgeries, chronic medical conditions such as (chronic heart failure, nephrotic syndrome, chronic lung diseases), known thrombophilia, malignancy-related factors, hormonal therapy, and prolonged immobilization [1]. Given the lack of specificity in clinical presentation, the uncertainty in diagnosing acute PE remains high. Typical ECG findings in acute PE include sinus tachycardia, RBBB, right axis deviation (RAD), S1Q3T3, atrial tachycardia or fibrillation, and T wave inversion or ST depression in leads V1 to V4 (1). Intermittent or transient right bundle branch block (RBBB) can occur in various clinical situations, including acute coronary syndromes and other cardiac conditions [2]. It is rarely described in acute pulmonary embolism [3]. The treatment of PE depends mainly on anticoagulation. However, in massive PE, patients may require thrombolytic therapy. Cardiopulmonary supportive treatment with oxygen therapy, mechanical ventilation, and inotropic agents is sometimes crucial [4, 5].

This case report focuses on a transit passenger who developed transient RBBB and transient S1Q3T3 pattern due to acute PE during EMS transport only, and he suffered maxillofacial trauma due to a syncopal episode. The EMS ECG provided the major clue to the diagnosis. The patient’s clinical picture was difficult to diagnose due to the language barrier, syncope, concurrent mild head injury, and maxillofacial trauma.

## Case presentation

**Figure 1:**
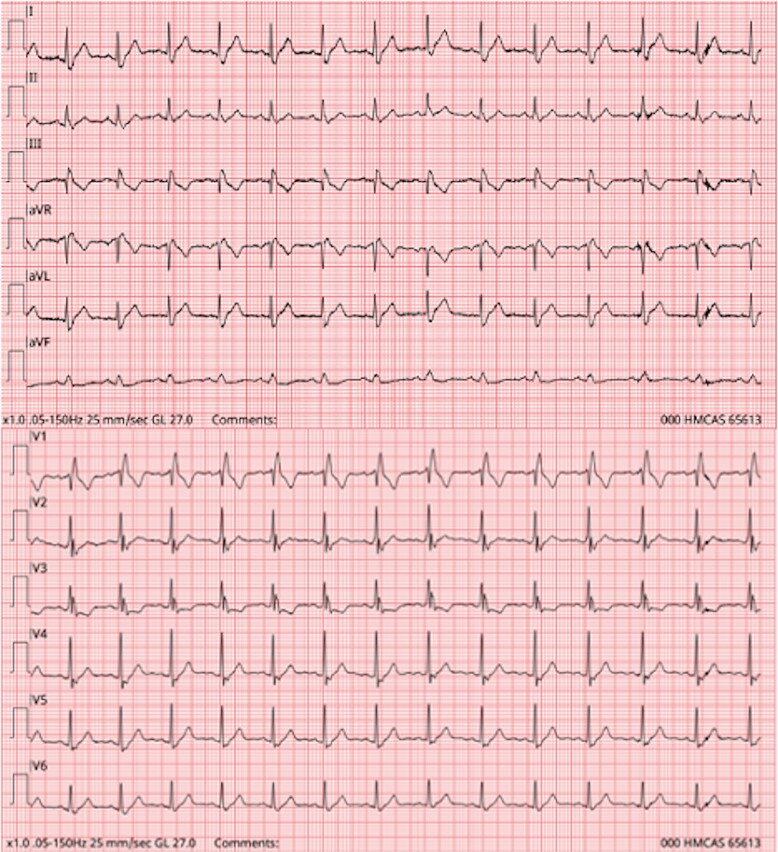
Shows EMS ECG showing right heart strain pattern with RBBB and S1Q3T3 pattern.

**Figure 2:**
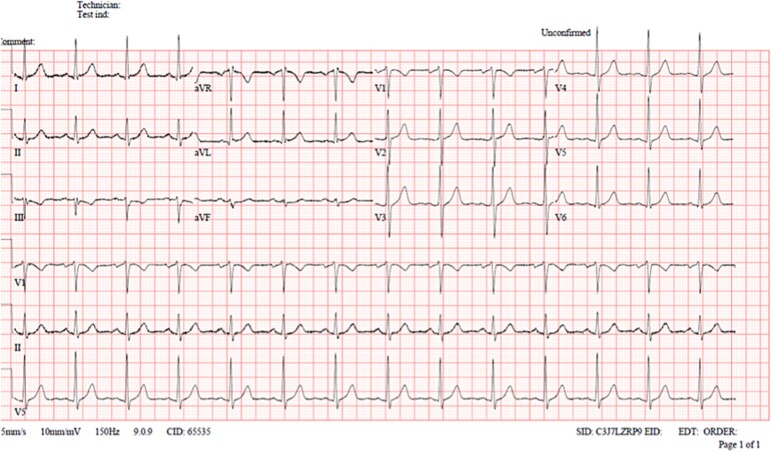
Shows normal ECG after the RV strain disappeared.

**Figure 3:**
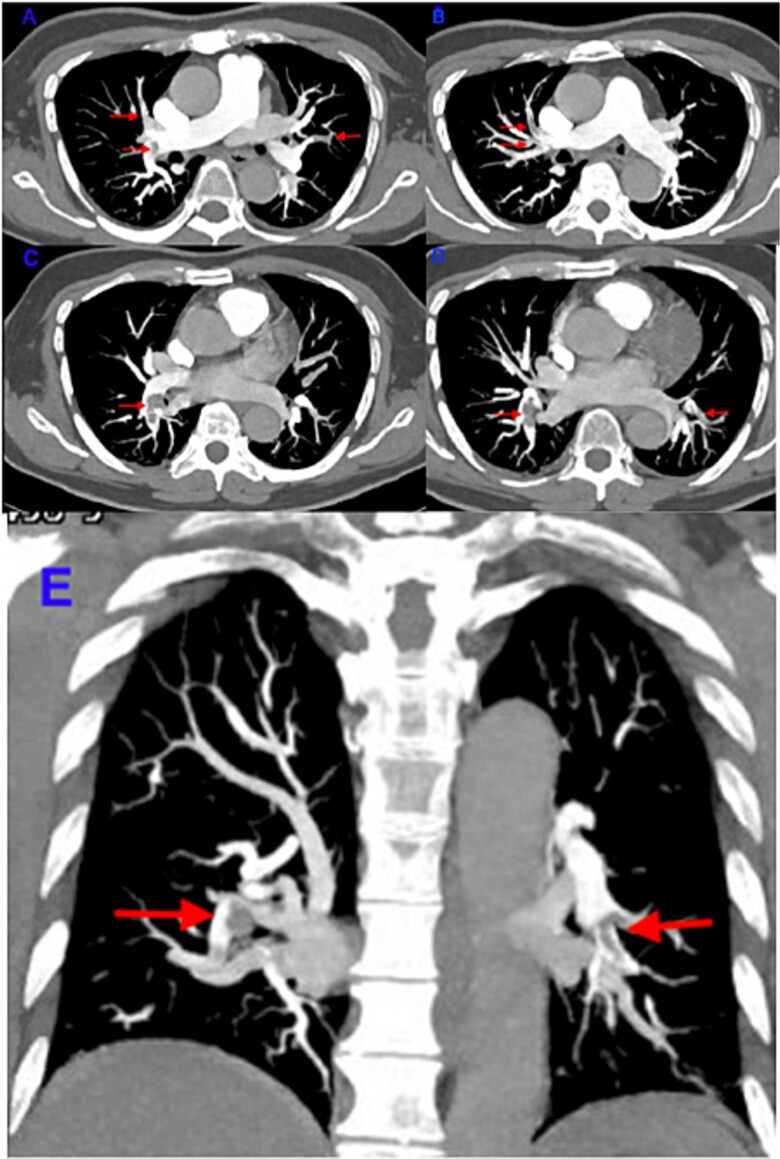
(A,B,C,D,E) shows filling defects in CT pulmonary angiogram.

A 57-year-old male transit passenger suffered a syncopal episode while boarding a connecting flight. He suffered a laceration on the left forehead scalp during the episode. Although language barriers initially impeded comprehensive history-taking, the patient denied any prior medical conditions. As per EMS, his initial saturations were 88% on room air. EMS brought him on eight liters via a non-rebreather mask. In the emergency department, vital signs were heart rate 81 bpm, respiratory rate 17 b/min, blood pressure 178/110 mmHg, and oxygen saturation 100% on room air. Physical examination revealed left frontal scalp laceration, left chin bruise, and intraoral laceration. An initial ECG performed by EMS demonstrated an RBBB with an S1Q3T3 pattern (Fig. 1A and B). However, subsequent ED ECGs were normal without RBBB and S1Q3T3 (Fig. 2). Bedside ultrasound (POCUS) of the heart showed normal contractility with no RV dilation. Laboratory values showed high D-dimer, high Troponin and high inflammatory markers (Table 1). The patient underwent a computed tomography pulmonary angiogram (CTPA), showing acute PE with filling defects in the right main pulmonary artery, its branches, the descending left pulmonary artery, and one of the upper lobe branches (Fig. 3A–E). Additionally, a plain CT head scan confirmed fractures involving the right maxillary sinus, right orbital floor, and nasal bones. Echocardiography showed normal right ventricle size, mildly reduced RV function, and a mild increase in pulmonary artery pressure.

**Table 1:** Pertinent lab values summary.

	Initial Laboratory values	After 48 hrs	Reference values
WBC	11.1	13.9	4–10 (10x10^3^/uL)
Absolute Neutrophil Count (ANC)	9.4	11.7	2.0–7.0 (10x10^3^/uL)
Hb	14.7	15.0	13–17 (gm/dl)
Platelets	163	192	150–410
PT	13.3	12.3	9.4–12.5 (seconds)
INR	1.1	1.0	0.9–1.1
APTT	41.9	32.6	25.1–36.5 (seconds)
D-dimer	22.87	___	0.00–0.49 (mg/L)
Sodium	139	138	133–146 (mmol/L)
Potassium	4.3	4.9	3.5–5.3 (mmol/L)
Bicarbonate	27	27	22–29 (mmol/L)
Urea	4.2	3.1	2.5–7.8 (mmol/L)
Creatinine	77	70	62–106 (umol/L)
AST	29	23	0–40 (U/L)
ALT	26	20	0–41 (U/L)
ALP	82	75	40–129 (U/L)
T. Bilirubin	8	16	0–21 (umol/L)
NT pro-BNP	82	___	<125(pg/mL)
CRP	20	___	
Troponin-T	154	___	3–15(ng/L)
Glucose	6.2	5.7	(3.3–5.5)
Ethanol	<2.2	______	Critical high>44.9 (mmol/L)

WBC = White Blood cell; Hb = Hemoglobin; PT = Prothrombin time; INR = International normalized ratio; aPTT = Activated Partial Thromboplastin Time; AST = Aspartate Aminotransferase; ALT = Alanine aminotransferase; ALP = Alkaline Phosphatase; T.Bi. =Total Bilirubin; CRP: C-Reactive protein.

The patient received an IV heparin bolus followed by infusion due to concerns about the possibility of increasing epistaxis due to facial fractures. Medical consultation led to admission. Maxillofacial and ophthalmology teams recommended conservative management for the facial and orbital fractures. The patient’s hospitalization course was uneventful. Anticoagulation therapy transitioned from IV heparin to enoxaparin and subsequently to oral rivaroxaban. He remained hospitalized for four days and was advised to avoid air travel for 7–10 days from symptom onset. A follow-up plan was established for the patient’s home country, encompassing assessment of fractures and further evaluation for venous thromboembolism.

## Discussion

This case is noteworthy for the transient nature of the RBBB and S1Q3T3 pattern observed in this patient’s initial ECG. Transient RBBB, although rare, has been associated with acute PE and is thought to result from acute strain on the right ventricle. The patient had likely developed a saddle embolism initially that caused an acute right ventricular strain pattern on ECG. The embolus might have traveled distally, which relieved the strain and reverted the ECG findings. It is important to recognize such ECG changes in the context of PE diagnosis.

RBBB is an ECG pattern that occurs due to interruption of normal electrical conduction through his-Purkinje fibers of the right ventricle. It is characterized by QRS duration ≥0.12 seconds, a secondary R wave (R) in V1 or V2, and a wide-slurred S wave in leads I, V5, and V6, often with associated ST-segment depression and T-wave inversion in the right precordial leads. It’s usually considered a benign finding; however, in many cases, it can be caused by hypertension, ischemic heart diseases, cardiomyopathy, valvular heart diseases, congenital heart diseases, pulmonary embolism, pulmonary hypertension, and Bragada syndrome [6]. A meta-analysis proposed that RBBB is associated with an increased risk of mortality in the general population and patients with heart disease. The pooled adjusted hazard ratio (HR) for all-cause mortality was 1.17 (95% confidence interval [CI]: 1.03–1.33) compared with no RBBB [7].

Studies have also indicated that ECG findings can help establish PE diagnoses and predict patients’ prognoses. A systematic review and meta-analysis proposed that the frequently encountered ECG features of PE are T wave inversion in V1 (38%), S1Q3TQ3 (24%), incomplete RBBB (12%), complete RBBB (10%), ST elevation in AVR (36%) [8].

Acute PE has been historically and is still considered a highly morbid condition that requires an immediate diagnosis and treatment. Multiple options of management are available, such as anticoagulation therapy, thrombolysis, catheter-directed therapy, and surgical embolectomy in patients with sub-massive and massive PE [5].

## Conclusion

This report highlighted a unique case involving acute PE, maxillofacial trauma, and transient RBBB with S1Q3T3 in a transit passenger with a language barrier. The rarity of transient RBBB and transient S1Q3T3 underscores the significance of its recognition as a potential sign of acute PE.
